# Quantifying the spatial patterns of retinal ganglion cell loss and progression in optic neuropathy by applying a deep learning variational autoencoder approach to optical coherence tomography

**DOI:** 10.3389/fopht.2024.1497848

**Published:** 2025-02-03

**Authors:** Jui-Kai Wang, Brett A. Johnson, Zhi Chen, Honghai Zhang, David Szanto, Brian Woods, Michael Wall, Young H. Kwon, Edward F. Linton, Andrew Pouw, Mark J. Kupersmith, Mona K. Garvin, Randy H. Kardon

**Affiliations:** ^1^ Center for the Prevention and Treatment of Visual Loss, Iowa City VA Health Care System, Iowa City, IA, United States; ^2^ Department of Ophthalmology and Visual Sciences, University of Iowa, Iowa City, IA, United States; ^3^ Department of Electrical and Computer Engineering, University of Iowa, Iowa City, IA, United States; ^4^ Iowa Institute for Biomedical Imaging, University of Iowa, Iowa City, IA, United States; ^5^ Department of Neurology, Icahn School of Medicine at Mount Sinai, New York, NY, United States; ^6^ Department of Ophthalmology, University Hospital Galway, Galway, Ireland; ^7^ Department of Physics, School of Natural Sciences, University of Galway, Galway, Ireland; ^8^ Department of Ophthalmology, Icahn School of Medicine at Mount Sinai, New York, NY, United States; ^9^ Department of Neurosurgery, Icahn School of Medicine at Mount Sinai, New York, NY, United States

**Keywords:** variational autoencoder (VAE), glaucoma, optic neuritis (ON), non-arteritic anterior ischemic optic neuropathy (NAION), retinal ganglion cell (RGC) loss, optical coherence tomography (OCT)

## Abstract

**Introduction:**

Glaucoma, optic neuritis (ON), and non-arteritic anterior ischemic optic neuropathy (NAION) produce distinct patterns of retinal ganglion cell (RGC) damage. We propose a booster Variational Autoencoder (bVAE) to capture spatial variations in RGC loss and generate latent space (LS) montage maps that visualize different degrees and spatial patterns of optic nerve bundle injury. Furthermore, the bVAE model is capable of tracking the spatial pattern of RGC thinning over time and classifying the underlying cause.

**Methods:**

The bVAE model consists of an encoder, a display decoder, and a booster decoder. The encoder decomposes input ganglion cell layer (GCL) thickness maps into two display latent variables (dLVs) and eight booster latent variables (bLVs). The dLVs capture primary spatial patterns of RGC thinning, while the display decoder reconstructs the GCL map and creates the LS montage map. The bLVs add finer spatial details, improving reconstruction accuracy. XGBoost was used to analyze the dLVs and bLVs, estimating normal/abnormal GCL thinning and classifying diseases (glaucoma, ON, and NAION). A total of 10,701 OCT macular scans from 822 subjects were included in this study.

**Results:**

Incorporating bLVs improved reconstruction accuracy, with the image-based root-mean-square error (RMSE) between input and reconstructed GCL thickness maps decreasing from 5.55 ± 2.29 µm (two dLVs only) to 4.02 ± 1.61 µm (two dLVs and eight bLVs). However, the image-based structural similarity index (SSIM) remained similar (0.91 ± 0.04), indicating that just two dLVs effectively capture the main GCL spatial patterns. For classification, the XGBoost model achieved an AUC of 0.98 for identifying abnormal spatial patterns of GCL thinning over time using the dLVs. Disease classification yielded AUCs of 0.95 for glaucoma, 0.84 for ON, and 0.93 for NAION, with bLVs further increasing the AUCs to 0.96 for glaucoma, 0.93 for ON, and 0.99 for NAION.

**Conclusion:**

This study presents a novel approach to visualizing and quantifying GCL thinning patterns in optic neuropathies using the bVAE model. The combination of dLVs and bLVs enhances the model’s ability to capture key spatial features and predict disease progression. Future work will focus on integrating additional image modalities to further refine the model’s diagnostic capabilities.

## Introduction

Glaucoma, optic neuritis (ON), and non-arteritic anterior ischemic optic neuropathy (NAION) are distinct ocular diseases that impact the optic nerve, causing structural damage within the retinal ganglion cell layer (1–5) (GCL). Glaucoma leads to progressive optic neuropathy and GCL thinning, characteristically of the superior and inferior optic nerve axon bundles with a higher frequency of inferior bundle involvement (2, 6, 7). ON, frequently linked to multiple sclerosis, is an inflammatory demyelinating condition causing optic nerve damage and global GCL thinning (1, 4, 8). NAION, a vascular disorder, results in acute damage to the optic nerve head (ONH), and disproportionately causes thinning of the superior optic nerve bundles (1, 3, 9). Each condition causes different spatial patterns of GCL (the layer containing cell bodies of the optic nerve bundles) defects, reflecting the corresponding pathophysiological mechanism and the retinal neurons most susceptible to damage. Therefore, GCL becomes an ideal target for spatial pattern analysis of disease-associated changes. Automating the quantification of GCL spatial patterns and systematically analyzing these patterns in a statistically meaningful manner provides a novel approach to improving the accuracy of optic neuropathy diagnosis and monitoring disease progression.

**Figure 1:**
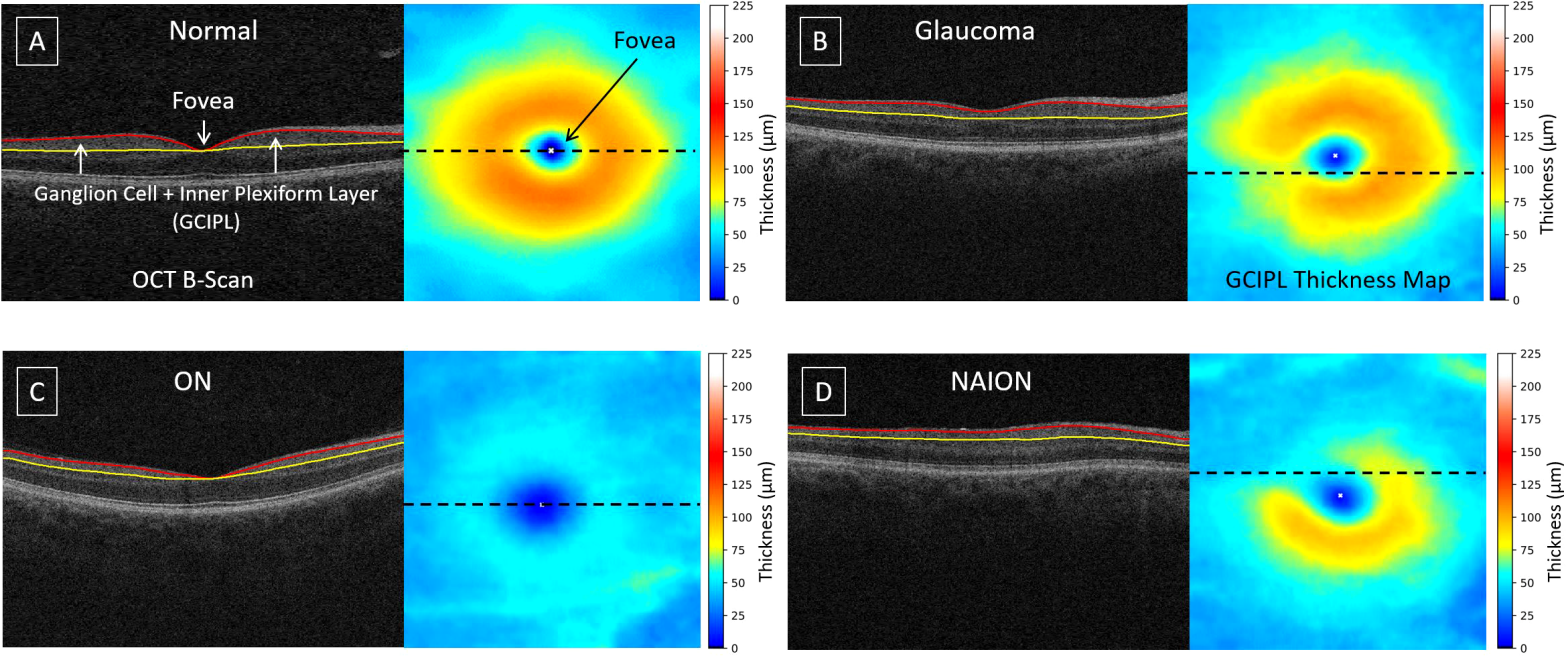
Examples of OCT B-scans with layer segmentation and their corresponding GCIPL thickness maps: **(A)** normal, **(B)** glaucoma, **(C)** optic neuritis, and **(D)** non-arteritic anterior ischemic optic neuropathy. The dashed line on the thickness map indicates the location of the B-scan. The GCIPL, defined as the region between the red and yellow surfaces, includes the ganglion cell and inner plexiform layers. The white 'x' marks the fovea center.

Optical coherence tomography (OCT) is a powerful tool for quantifying optic nerve damage in ocular diseases (1, 5). OCT provides cross-sectional retinal information, enabling quantitative assessments of retinal layers. Advanced layer segmentation techniques facilitate the creation of the ganglion cell plus inner plexiform layer (GCIPL) thickness maps (10–13), which are essential for identifying and tracing optic nerve damage. **Figure 1** presents examples of OCT B-scans with layer segmentation and the corresponding GCIPL thickness maps. In a normal retina (**Figure 1A**), the highest density of ganglion cell bodies and their dendritic connections are typically located 4 – 6 degrees away from the fovea center, forming an annular pattern (14). When optic nerve bundles are injured in conditions like glaucoma, ON, or NAION (**Figures 1B–D**), retinal ganglion cells and their axons degenerate, leading to localized GCIPL thinning. The pattern of thinning corresponds to the spatial patterns of axon loss, providing a link between optic nerve damage and changes in GCIPL thickness/patterns. This relationship is beneficial for estimating the damaged regions by automated approaches.

Variational autoencoders (15) (VAEs) are a type of deep-learning generative model and have been widely applied to disentangle image features and create synthetic images that morph among objects (16–19). During training, the VAE encoder decomposes input images into lower-dimensional latent variables (LVs), while the co-trained decoder reconstructs the input images from these LVs. Previous studies have demonstrated that VAE models can be used for ophthalmic images in the cornea or retina and for different diseases (20–28) (e.g., keratoconus and glaucoma). Recently, we proposed a customized VAE model to visualize the glaucomatous GCL thinning patterns in OCT using only two LVs and still maintaining smooth pattern transitions in two-dimensional (2D) latent space (LS) montage maps (6). We further refined our VAE design to categorize OCT-based ONH optic nerve swelling patterns in the LS for various severities of papilledema, based on the Frisén grades provided by experienced neuro-ophthalmologists (29). These studies indicate that VAE models are flexible and have great potential to synthesize and integrate meaningful patterns of optic nerve damage from multiple diseases.

In this study, we propose a booster VAE (bVAE) model that computes two display latent variables (dLVs) to synthesize meaningful optic nerve patterns organized in a 2D LS montage map, capturing statistically meaningful GCL spatial patterns for glaucoma, ON, and NAION. Eight booster latent variables (bLVs) are designed and employed to encode additional features/details beyond which two dLVs can represent. We also incorporate specialized loss functions to encourage the LS to reflect the natural transition of GCL thinning patterns. Furthermore, machine learning classifiers [XGBoost (30)] are utilized to leverage both dLVs and bLVs to identify the GCL thinning and classify the cause. This study establishes a foundational framework for organizing GCL spatial patterns across three prevalent optic neuropathies. The identified GCL patterns serve as anchor points for a future unified latent space, enabling the feasible integration of images from different locations in the retina (e.g., ONH and/or widefield OCT) and modalities (e.g., visual fields, color fundus photos, OCT texture images, and OCT angiography) for comprehensive disease analysis.

## Materials and methods

### Overview

**Figure 2:**
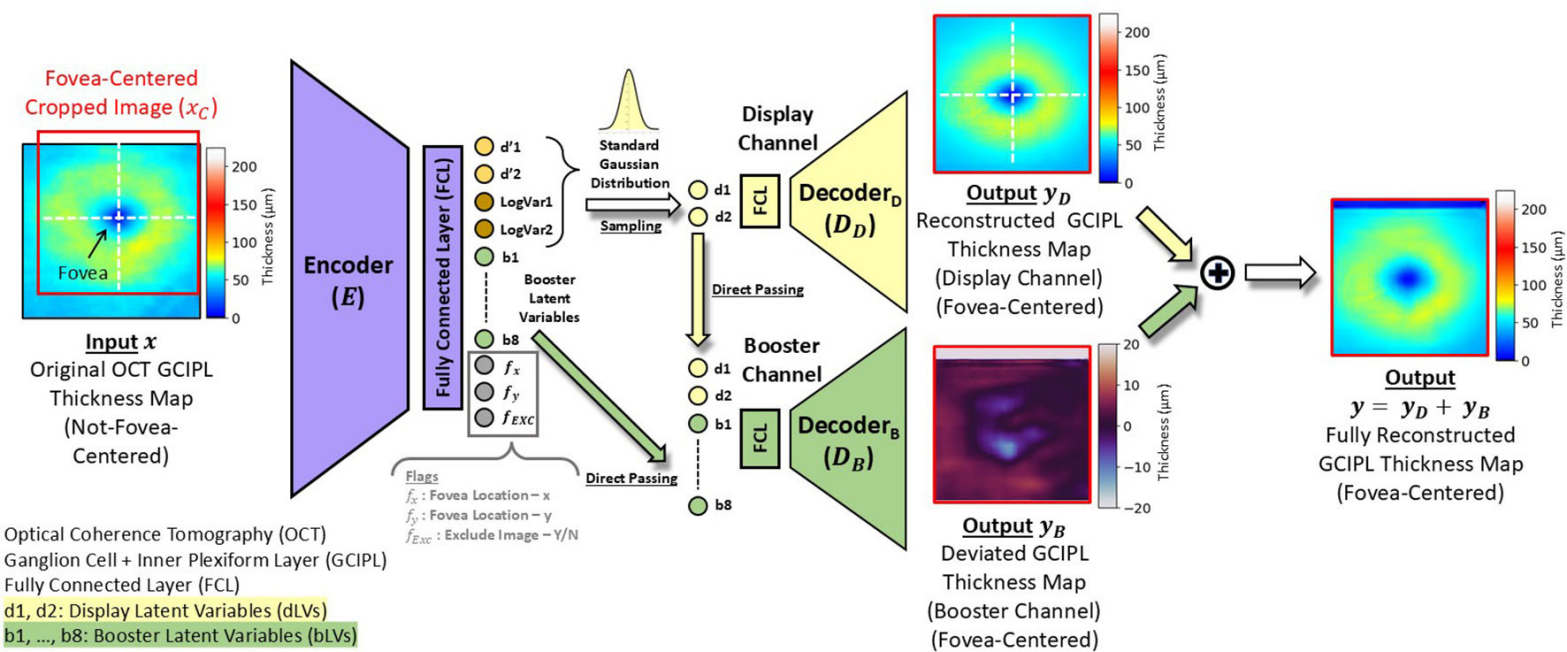
Flowchart of the proposed booster variational autoencoders (bVAE) model.

**Figure 3:**
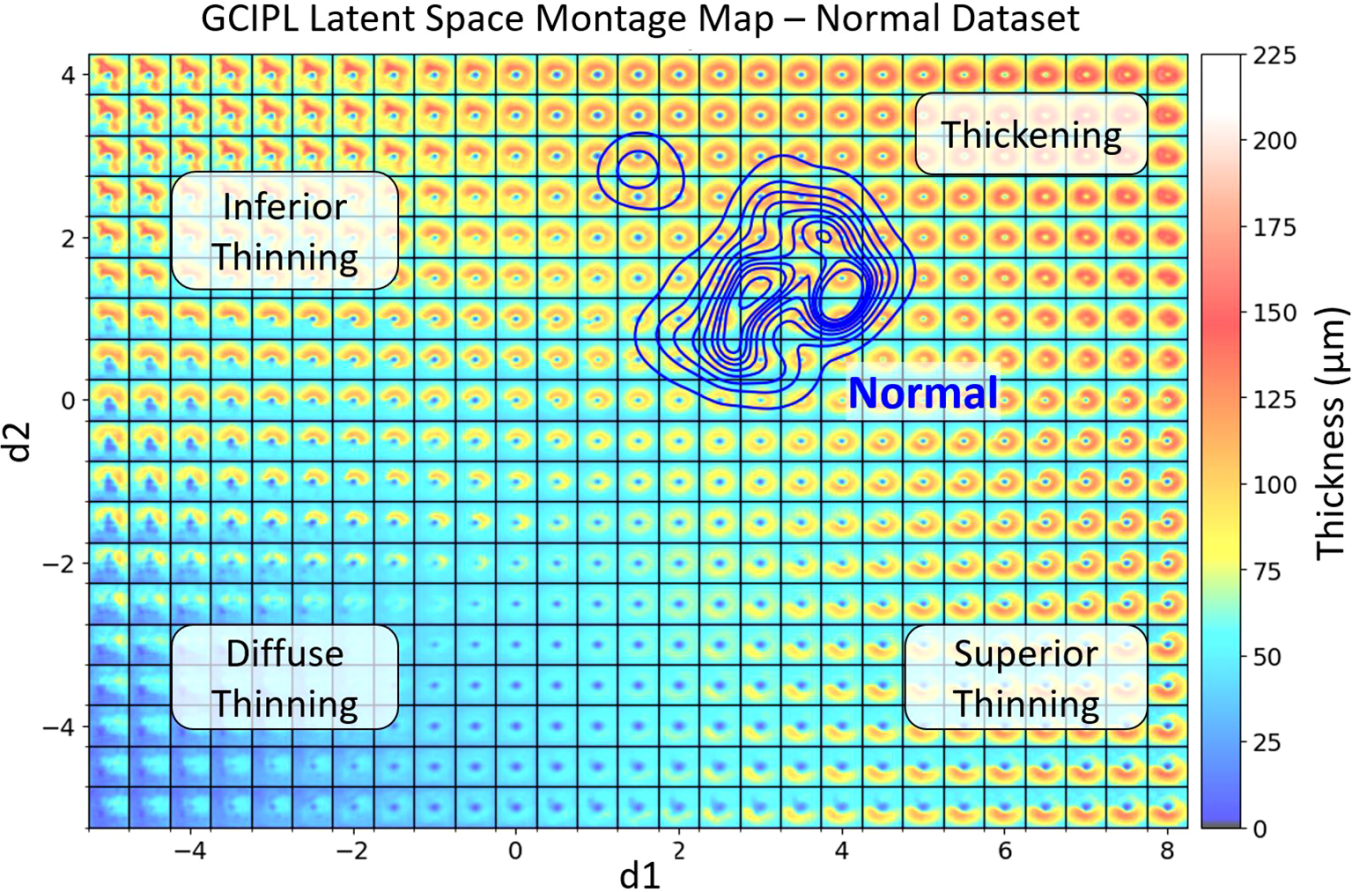
The generated GCIPL latent space (LS) montage map shows the spatial patterns of GCIPL thickness generated by the bVAE model, utilizing both display and binary latent variables (dLV’s and bLV’s). The LS was trained based on a training set of GCIPL thickness maps from normal eyes and eyes with glaucoma, optic neuritis and NAION. Each spatial pattern tile is defined by its latent variables. The two main display latent variables d1 and d2 are plotted on the x and y axis, respectively. The blue KDE (32) contour lines represent the distribution of the Iowa normal dataset, defining a normal range of spatial patterns encountered. Regions of the latent space corresponding to inferior, diffuse, and superior thinning are labeled. When an individual patient eye’s GCIPL thickness map is derived from the bVAE model, it can be plotted onto this LS montage map. This allows one to immediately visualize how normal or abnormal the pattern is and its location on the montage provides information on the spatial pattern (e.g. thinning in the superior or inferior retina or diffuse thinning). Plotting multiple time points also allows one to track whether the spatial pattern is changing over time to assess progression of disease (examples shown in **Figure 5**). The extreme top-left region of the montage demonstrates an unusual spatial pattern of GCIPL thickness not due to optic nerve damage, which was influenced by a patient’s eye in the training set having an epiretinal membrane, which distorted the spatial pattern of the segmented GCIPL layer.

**Figure 2** illustrates the framework of the proposed bVAE model, comprising an encoder, a display decoder, and a booster decoder. After training, the display decoder can synthesize GCIPL thickness maps from input dLV pairs, which can be organized to form the LS montage map (**Figure 3**), displaying meaningful and statistical GCIPL spatial patterns.

### Data

This study includes four independent datasets. For the normal and glaucoma groups, data were obtained from 66 and 189 subjects, with a total of 937 and 4,192 macular OCT scans, respectively, from the University of Iowa Hospitals and Clinics. The NAION dataset comprised 351 subjects with 2,957 OCT macular scans, sourced from a subset of the Quark Pharmaceutical Clinical Trial (31) (ClinicalTrials.gov Identifier: NCT02341560). The ON dataset included 216 subjects with 2,615 OCT macular scans from New York Mt. Sinai Hospital. This optic neuritis dataset only included patients with typical optic neuritis and did not include those with myelin oligodendrocyte glycoprotein antibody-associated disease (MOGAD) or neuromyelitis optica (NMO) optic neuritis. Each subject had macular OCT volumetric scans available for either single or both eyes, with data collected from a single or multiple visits. For each disease, 10 and 25 subjects were randomly selected for the validation and test sets, respectively; no OCT scans from the same subjects were included across the training, validation, and test sets. To ensure the VAE model was exposed to a wide range of GCIPL patterns, all available OCT scans (including both good and bad scans; details of image quality control are provided in the **Supplementary Document**) in the training set were included in the training process. The validation dataset was used to help decide when the model should stop training. The test dataset were used to evaluate reconstruction errors and for estimation of the spatial patterns of GCL thinning over time. The study was approved by the Institutional Review Boards (IRBs) of the University of Iowa and the Mount Sinai School of Medicine and adhered to the tenets of the Declaration of Helsinki.

There were a total of 10,701 OCT macular volumetric scans, of which 10,686 of these scans were Cirrus scans (Carl Zeiss Meditec, Dublin, CA) covering 6 × 6 × 2 mm³. The Cirrus device employed two protocols: one with 200 B-scans (200 × 1024 pixels) and another with 128 B-scans (512 × 1024 pixels). Additional 15 scans from the normal dataset were obtained using the Optovue XR Avanti System (Visionix USA, Inc., Lombard, IL), also covering 6 × 6 × 2 mm³, with a protocol of 400 B-scans (400 × 640 pixels). After layer segmentation, all GCIPL thickness maps were resized to 200 × 200 pixels for the input into the bVAE model.

It is worth noting that, as in real-world clinical practice, the populations of subjects with healthy nerves or glaucoma, ON, and NAION in these datasets have differences in demographic composition. Although age and race may influence overall GCIPL thickness, these factors may have limited effects on the anatomical spatial patterns of the GCIPL. The bVAE model employs an unsupervised framework to organize GCIPL thickness maps in LS based solely on spatial pattern similarity. More details are provided below in the bVAE model design.

### Ganglion cell + inner plexiform layer thickness maps

For each OCT macular scan, the GCIPL was automatically segmented using our hybrid deep learning algorithm [Layered Optimal Graph Image Segmentation for Multiple Objects and Surfaces; Deep LOGISMOS (10)]. Following segmentation, all GCIPL thickness maps underwent quality control by an OCT expert (J-KW). This process involved correcting any errors in the automated identification of the fovea location and flagging thickness maps with unacceptable image quality, such as those where segmentation failed due to poor OCT signal strength. Any cases that failed quality control were flagged. Also, despite the use of three OCT scan protocols in our data, all OCT scans cover the same physical dimensions with adequate numbers of B-scan to render a volume scan, leading to consistent GCIPL thickness map quality across the dataset and across different OCT instrument manufactures. Examples of typical layer segmentation and GCIPL thickness maps for normal, glaucoma, ON and NAION cases are shown in **Figure 1**.

Next, for each eye with more than three visits, the GCIPL thickness maps that passed quality control were reviewed longitudinally to assess changes over time. The presence or absence of GCIPL thinning was determined based on visual inspection. Each eye was then assigned one of two labels: GCIPL thickness changes beyond the normal range over time or the changes that remained within the normal range. Details on data quality control and the visual inspection of GCIPL thickness changes are provided in the **Supplementary Document**.

### Booster variational autoencoders

In this study, we propose a novel VAE architecture (**Figure 2**), referred to as the booster VAE (bVAE), by modifying our previous bi-channel VAE model, which was initially designed for determining the patterns and changes in ONH swelling due to papilledema (29). This new bVAE consists of an encoder (*E*), a display decoder ( $D_{D}$ ), and a booster decoder ( $D_{B}$ ), which are co-trained but do not share parameters. After training, the encoder E decomposes an input GCIPL thickness map ( x ) into two display latent variables (dLVs; d1 and d2) and eight booster latent variables (bLVs; b1 – b8). Next, $D_{D}$ reconstructs a fovea-centered cropped version of the input ( $x_{C})$ using only the two dLVs (d1 and d2), producing a fovea-centered reconstructed map ( $y_{D}$ ). Simultaneously, the booster decoder $D_{B}$ utilizes all latent variables (d1, d2, and b1 – b8) to enhance the reconstruction by compensating for the display decoder and adding finer details ( $y_{B}$ ), resulting in a more accurate final output ( $y=y_{D}+y_{B}$ ).

Although the OCT scan protocol focused on the macula, the images were not always perfectly centered at the fovea (e.g., x in **Figure 2**). During training, we designed specific reconstruction loss functions to ensure that the outputs from both decoders ( $y_{D}$ and $y_{B}$ ) are fovea-centered. This design allows the reconstructed GCIPL thickness maps to be directly used for further machine-learning tasks without the need for additional alignment steps. Additionally, the encoder estimates the fovea location ( $f_{x}$ and $f_{y}$ ) and generates a flag ( $f_{EXC}$ ) to identify the input GCIPL thickness maps with poor image quality.

An important feature of our bVAE model is the customization of the dLVs to reflect the GCIPL annulus patterns. Along with Kullback-Leibler (K-L) divergence (15) to regulate the dLVs during training, we applied additional penalties to d1 and d2 based on the ratio of GCIPL annulus thickness in the inferior and superior regions, respectively. This design guided the 2D LS to follow a predefined organization: top-left for inferior thinning, top-right for normal and thickening, bottom-left for diffuse thinning, and bottom-right for superior thinning (**Figure 3**). Notably, no clinical information (e.g., disease labels) was used during training, so the bVAE remains unsupervised to disease classifications. Further details on loss function design are provided in the **Supplementary Methods**.

### Latent space montage map visualization

After training, each available GCIPL thickness map was encoded into a data point in the 2D LS montage map, organized by the display latent variables (dLVs; d1 and d2) that capture major spatial patterns of GCIPL thinning. Disease labels corresponding to the data points are color-coded and displayed in the LS montage map, providing a visualization of spatial patterns associated with specific optic neuropathies. The normal distribution of data points is outlined by kernel density estimation (KDE) contours, generated using a Gaussian kernel to create a smooth boundary around the data points’ locations [Python package Seaborn (32)]. By plotting individual scans or longitudinal trajectories onto this map, researchers can observe progression over time, compare stages across diseases, and assess spatial deviations from normative patterns. This setup offers an intuitive tool for diagnostic insights and disease progression monitoring.

### Classification of GCIPL thinning status

Because the primary focus of this study is the bVAE model and its LS display and booster latent variables (dLVs and bLVs), any standard machine learning classifier could be applied to use these latent variables as features to classify GCIPL thinning status. For this study, we selected XGBoost (30), which is a gradient-boosting algorithm well-suited for structured data. XGBoost incorporates multiple techniques to prevent overfitting, including L1 and L2 regularization, tree pruning, subsampling, and a shrinkage parameter to control the learning rate. XGBoost also provides feature importance metrics, which could aid future studies in evaluating the influence of each latent variable on thinning status classification.

### Model evaluations

To assess the reconstruction errors among the original (and the fovea-centered version, $x_{C}$ ), dLV reconstructed (*y_D_*), and fully reconstructed ( y ) GCIPL thickness maps, three metrics were employed to evaluate the proposed bVAE’s performance from different perspectives. The image-based root-mean-square error (RMSE) was first included, because it is a common metric to determine how closely a generative model can reconstruct the input images. However, RMSE can be sensitive to existing noise or artifacts in the input images. In our case, the GCIPL thickness maps may not be fovea-centered or contain non-neuropathy-related features (e.g., epiretinal membrane or vessel shadows). Therefore, the structural similarity (SSIM) index (33, 34), ranging from 0 (no similarity) to 1 (identical images), was also included to quantify structure changes between $x_{C}$ and reconstructed images ( $y_{D}$ and y ). Additionally, sectorial thicknesses in a GCIPL elliptical annulus grid were computed to provide a clinically meaningful evaluation metric. This annulus grid covers the same area as Zeiss Cirrus software analyzing GCIPL thickness with a vertical inner and outer radius of 0.5 and 2.0 mm; and a horizontal inner and outer radius of 0.6 and 2.4 mm, respectively. The annulus was further divided into six sectors, which include superior nasal (SN), superior (S), superior temporal (ST), inferior temporal (IT), inferior (I), and inferior nasal (IN) sectors.

To evaluate the ability of dLVs and bLVs to identify spatial patterns of GCIPL thinning beyond the normal range over time, XGBoost (30) was used for classification, with the dLVs alone or both dLVs and bLVs as input features from three time points. The first time point was chosen from the first visit, and the third time point was from the last visit. The second time point was chosen as the visit most equidistant from the first and last visits when there were more than three visits. The XGBoost parameters were optimized by using our training and validation datasets, with a maximum tree depth of 15, a step size shrinkage of 0.3, and “multi:softprob” as the objective to predict the probability of thinning. XGBoost classifications were performed in two experiments. The classifier estimated the probability of an eye being classified as showing a spatial pattern of GCIPL thinning beyond the normal range versus remaining within the normal range, based on the data from the first, second, and third time points. Second, instead of binary classification, the XGBoost model estimated the probability of each specific label (i.e., within normal range, glaucoma, ON, and NAION) for each eye across the same three visits. Multiclass Receiver Operating Characteristic (ROC) curves and area under the curve (AUC) were further utilized to assess performance based on the estimated probabilities for each eye in the independent test dataset.

## Results

**Table 1** presents the demographic and data distribution for the training, validation, and test sets, all of which were randomly selected at the subject level. The training set includes 692 subjects with a mean age of 56.14 years and a total of 8,934 OCT scans, which were all (including both good and bad scans) used for training the bVAE model. The validation set includes 30 subjects with a mean age of 54.99 years and 267 OCT scans, and the test set includes 100 subjects with a mean age of 51.94 years and 1,500 OCT scans. GCIPL thickness measurements, reported as the mean thickness across six sectors for scans passing quality control, are provided for each group.

**Table 1:** Demographics and data distribution of the training, validation, and test datasets.

		Training Set				
		Normal	Glaucoma	NAION	ON	Total
Subject Count		41	154	316	181	692
Age (Mean ± SD y.o.)		39.68 ± 19.43	65.68 ± 9.89	61.43 ± 7.74	42.65 ± 13.26	56.14 ± 14.61
Sex (M/F)		24/17	68/86	238/78	51/130	381/311
Race	White	39	144	211	119	513
	Black	2	7	1	13	23
	Asian	0	1	103	3	107
	Hispanic	0	2	0	5	7
	Other	0	0	1	41	42
Visit	Mean Number	4.27 ± 4.66	7.40 ± 3.03	4.07 ± 1.25	5.61 ± 4.13	5.22 ± 3.20
	Interval (Days)	126.02 ± 100.41	282.34 ± 270.24	65.48 ± 68.41	269.96 ± 328.12	194.19 ± 256.02
OCT Count		630	3451	2639	2214	8934
Good Scan (^†^GCIPL Thickness)		620 (78.68 ± 5.95)	3309 (65.48 ± 11.34)	2553 (72.03 ± 14.64)	2062 (71.29 ± 13.04)	8544 (69.79 ± 13.13)
^‡^Bad Scan		10	142	86	152	390
		Validation Set				
		Normal	Glaucoma	NAION	ON	Total
Subject Count		0	10	10	10	30
Age (Mean ± SD y.o.)		—	69.68 ± 7.93	56.50 ± 3.66	38.79 ± 16.17	54.99 ± 16.45
Sex (M/F)		—	8/2	5/5	4/6	17/13
Race	White	—	10	10	7	27
	Black	—	0	0	0	0
	Asian	—	0	0	1	1
	Hispanic	—	0	0	0	0
	Other	—	0	0	2	2
Visit	Mean Number	—	5.40 ± 2.99	4.10 ± 0.74	3.60 ± 2.91	4.37 ± 2.48
	Interval (Days)	—	383.41 ± 321.24	65.98 ± 65.72	289.69 ± 359.70	258.31 ± 310.93
OCT Count		0	111	82	74	267
Good Scan (^†^GCIPL Thickness)		—	83 (68.96 ± 10.51)	82 (77.73 ± 15.05)	74 (68.81 ± 9.46)	239 (71.92 ± 12.66)
^‡^Bad Scan		0	28	0	0	28
		Test Set				
		Normal	Glaucoma	NAION	ON	Total
Subject Count		25	25	25	25	100
Age (Mean ± SD y.o.)		38.41 ± 20.30	67.62 ± 9.66	57.56 ± 5.58	44.17 ± 12.78	51.94 ± 17.36
Sex (M/F)		17/8	9/16	16/9	6/19	48/52
Race	White	22	24	15	18	79
	Black	3	0	0	0	3
	Asian	0	0	9	1	10
	Hispanic	0	1	0	1	2
	Other	0	0	1	5	6
Visit	Mean Number	3.80 ± 4.65	7.40 ± 2.97	4.60 ± 1.53	6.00 ± 4.49	5.45 ± 3.84
	Interval (Days)	107.17 ± 91.14	273.84 ± 288.98	62.40 ± 65.87	256.12 ± 260.31	195.30 ± 239.50
OCT Count		307	630	236	327	1500
Good Scan (^†^GCIPL Thickness)		305 (80.86 ± 6.02)	610 (65.32 ± 12.45)	226 (71.05 ± 15.35)	310 (71.67 ± 11.67)	1451 (70.84 ± 13.13)
^‡^Bad Scan		2	20	10	17	49

^†^GCIPL Thickness represents the mean thickness (µm) across the six sectors of the GCIPL annulus for the corresponding dataset.

^‡^Bad scans are the OCT images with bad image quality, unsuccessful layer segmentation, fovea off-center, and/or incorrect labels.

**Figure 4:**
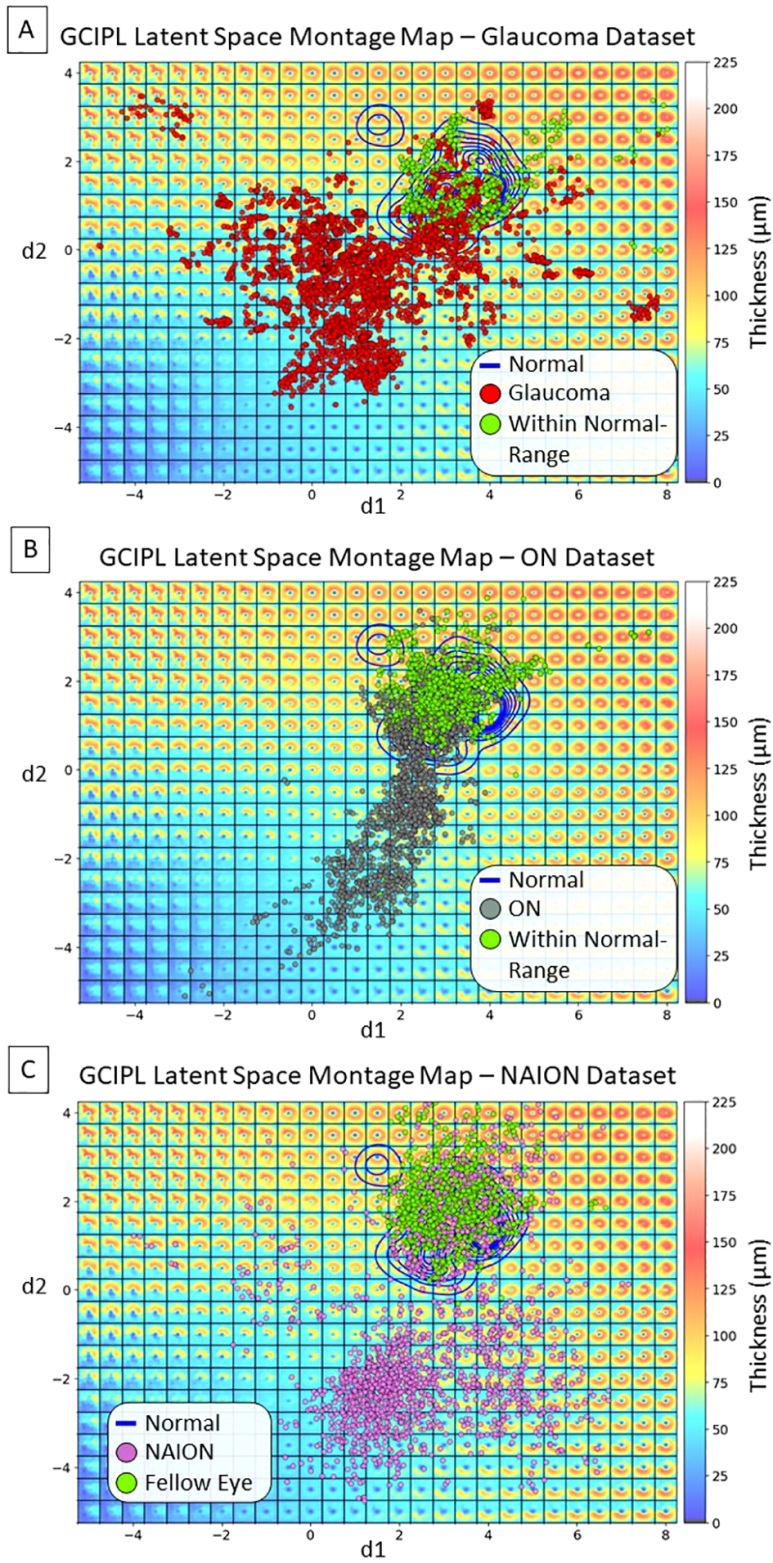
Distributions of display latent variables (dLVs) for all the available GCIPL thickness maps. **(A)** Glaucoma dataset: the red dots indicate thickness maps from the same eye showing thinning beyond the normal range over time, some of which started out in the normal range, while the green dots indicate thickness maps that remained within the normal range due to that eye having only very mild or no glaucomatous damage within the macula. **(B)** ON dataset: the gray dots indicate maps from the same eye showing thinning beyond the normal range over time, after starting out in the normal range; the green dots indicate thickness maps that remained within the normal range in either the affected eye or the fellow eye without optic neuritis. **(C)** NAION dataset: the purple dots indicate maps from eyes with NAION over time. At early time points there may be thickening due to optic disc edema and these eyes initially fell into the normal range or showed thickening, but over time the GCIPL became thin in the same eye; the green dots indicate maps from fellow eyes that had normal spatial patterns of GCIPL thickness.

**Figure 3** shows a GCIPL 2D latent space (LS) montage, where each tile was generated by inputting dLV pairs (d1, d2) ranging in values from (−5, −5) to (8, 4) . There were a total of 10,701 GCIPL thickness maps in our dataset, where 10,639 (99.42%) thickness map dLV pairs were in this range. Based on the model design, The LS montage map’s top-left, bottom-left, and bottom-right regions correspond to GCIPL inferior, diffuse, and superior thinning, respectively. The blue contour lines were drawn using the KDE plot [by the Python package Seaborn (32)] to indicate the distribution of the Iowa normal data points. Note that a glaucoma eye in the training set with repeated visits had an epiretinal membrane which produced an artifactual spatial pattern of segmented GCIPL thickness that was outside of the patterns encountered due to optic nerve disease. This resulted in the encoding of spatial patterns in the extreme upper left corner of the latent montage that did not correspond to thinning caused by optic nerve pathology. Next, **Figure 4** displays the distributions of all available data points for eyes from the glaucoma, ON, and NAION datasets, respectively. Each dot represents a GCIPL thickness map based on its dLV pair.

**Figure 5:**
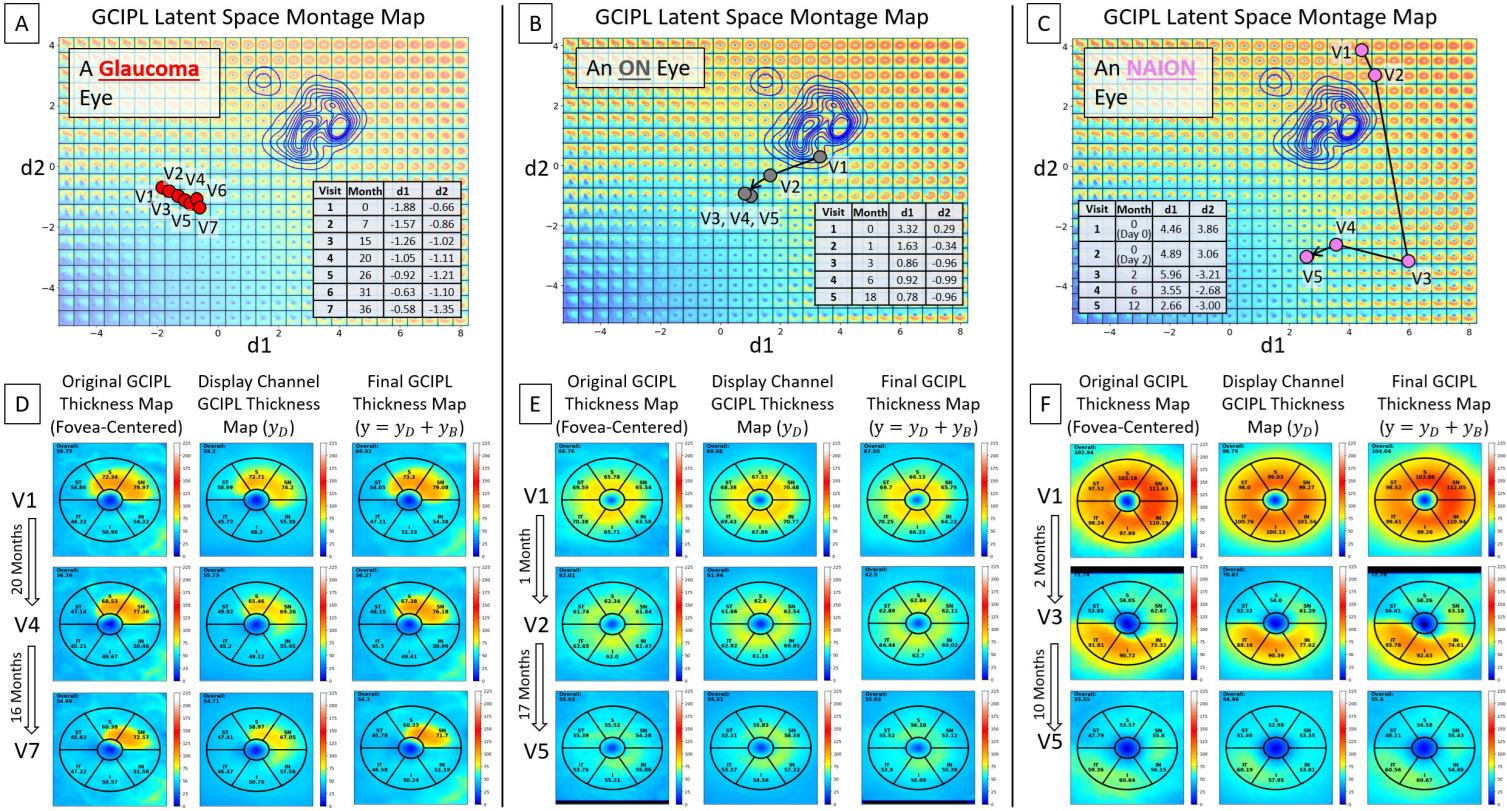
Visualization of longitudinal GCIPL thickness data using the VAE LS montage map. **(A–C)** Data trajectories for a glaucoma, ON, and NAION eye, with details between patient visits shown. **(D–F)** Original GCIPL thickness maps (the left columns), reconstructed maps using only two dLVs ( $y_{D}$ ; the middle columns), and fully reconstructed maps using both dLVs and bLVs ( y ; the right columns). The bVAE model captures the key features with only two parameters (dLVs) and closely approximates the input with just 10 parameters (dLVs and bLVs: d1, d2, b1 – b8).

Three examples are presented in **Figure 5**, utilizing the bVAE LS montage map to visualize GCIPL thickness data over time to assess progressive thinning. **Figures 5A–C** show the data trajectories for a glaucoma, ON, and NAION eye, respectively, with the time between patient visits (in months) indicated. By tracking the location of these trajectories within the LS montage maps, this novel bVAE LS visualization offers a comprehensive way to observe the progression of GCIPL changes over time due to different causes. **Figures 5D–F** display the original fovea-centered GCIPL thickness maps (left column), the reconstructed maps ( $y_{D}$ ; the middle column) using only the two dLVs (i.e., d1 and d2), and the fully reconstructed maps ( $y=y_{D}+y_{B}$ ; the right column) using both two dLVs and eight bLVs (i.e., b1 to b8). The reconstructed maps from dLVs alone ( $y_{D}$ ) demonstrate that the display decoder captured the main spatial patterns of GCIPL thinning. When both dLVs and bLVs were used, the final reconstructed map ( y ) closely approximated the original GCIPL thickness map, which captured finer spatial features. In other words, the bVAE could represent the GCIPL thinning patterns using only two parameters (dLVs: d1 and d2), while the full GCIPL reconstruction could be achieved using just 10 parameters (dLVs and bLVs: d1, d2, b1 to b8).

**Figure 6:**
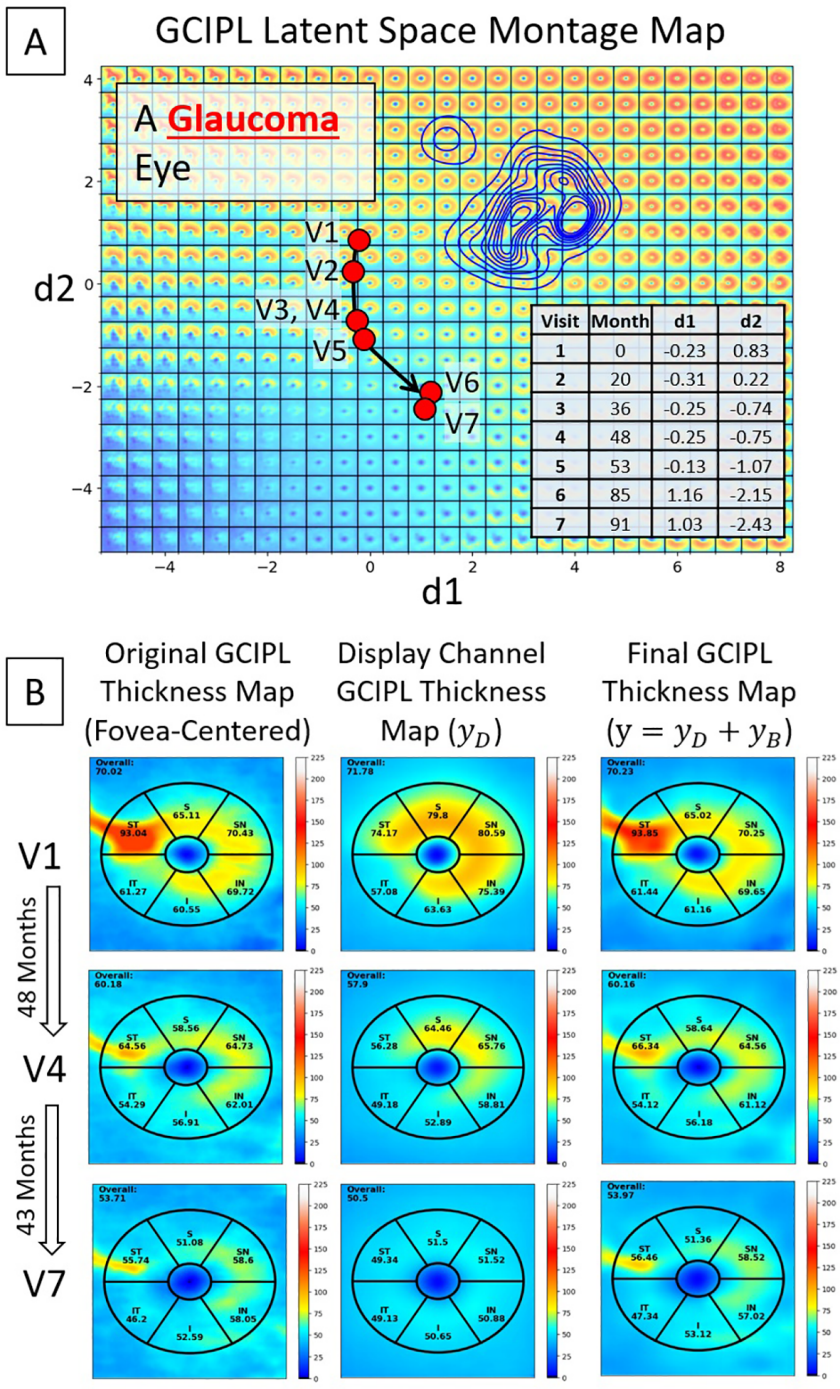
An example of a glaucoma eye that shows thickening in the superior temporal sector due to an epiretinal membrane. **(A)** A trajectory line of a glaucoma eye showing progression. **(B)** Original GCIPL thickness maps (the left column), reconstructed GCIPL thickness maps using only two dLVs (the middle column), and reconstructed GCIPL thickness maps using both dLVs and bLVs (the right column). Note that the dLVs minimize the impact of such anomalies, and the bLVs, which encode finer spatial details, compensate for the details not encoded by the two dLV’s, providing a means of encoding spatial information that is statistically less common. Differences between the thickness maps encoded by the two dLVs vs. the 10 latent variables (dLVs + bLVs) can reveal common artifacts, such as an epiretinal membrane in this example.

In a glaucoma eye example shown in **Figure 6**, an epiretinal membrane resulted in artifactual thickening in the superior temporal sector of the scan. The display bVAE encoder ignored this artifact due to its high spatial order characteristics, but the bLV model with 10 latent variables captured this feature. Comparing differences in the decoded spatial pattern using two dLVs *vs*. 10 variables (2 dLVs + 8 bLVs) can help reveal higher degree spatial features that may or may not be clinically useful, depending on the disease and how it is affected by pathology.

For the quantitative results, the bVAE encoder yielded an accuracy of 98.47% (sensitivity: 0.82 and specificity: 0.99) in identifying spatial patterns of GCIPL thickness maps with poor image quality in the independent test dataset that could be excluded. Among the 1,451 GCIPL thickness maps that passed quality control (**Table 1**), the bVAE encoder achieved a mean signed difference (i.e., estimated fovea location minus true fovea location) ± standard deviation of -0.05 ± 3.81 pixels in the x-direction and -0.27 ± 3.52 pixels in the y-direction.

To ensure that each eye was represented only once per visit, one OCT scan was randomly selected from each eye per visit, resulting in a total of 933 OCT scans from 179 eyes in the test dataset. The overall image-based RMSE between the original GCIPL thickness maps ( x ) and the dLV reconstructed maps ( $y_{D}$ ) was 5.55 ± 2.29 µm. With the inclusion of bLVs, the image-based RMSE improved to 4.02 ± 1.61 µm, which is significantly decreased ( p -value ≪ 0.001). For sectorial thickness estimation, compared to x , $y_{D}$ yielded an overall standard deviation of 1.80 µm across the entire Zeiss Cirrus GCIPL annulus grid. Specifically, the standard deviation of the thickness difference was 1.07 µm for the normal group, 2.69 µm for glaucoma, 1.24 µm for ON, and 1.36 µm for NAION. When bLVs were incorporated, the overall standard deviation improved to 0.94 µm across the same annulus grid, with successfully decreased variability across all neuropathy groups and sectors, indicating an enhancement in reconstruction accuracy with bLVs. Finally, SSIM index showed very similar mean and standard deviation results between x *vs*. $y_{D}$ and x *vs*. y for all neuropathy groups. This implies that while the fully reconstructed image y (incorporating bLVs) offered improved reconstruction accuracy compared to $y_{D}$ (which is only based on two dLVs), the SSIM index suggests that $y_{D}$ alone can capture the essential GCIPL spatial patterns effectively. **Table 2** provides detailed values for all comparisons.

**Table 2:** Evaluations of reconstructions between the original ( x ) and bVAE reconstructed GCIPL thickness maps (*y_D_* and *y*).

		Original ( x ) vs. dLVs Reconstructed GCIPL Maps ( $y_{D}$ )					Original ( x ) vs. dLVs+bLVs Reconstructed GCIPL Maps ( y )				
		Normal	G	ON	NAION	“All”	Normal	G	ON	NAION	“All”
**^†^RMSE** Mean (± Std)		4.17 (0.88)	6.84 (2.68)	4.41 (1.14)	6.37 (2.38)	5.55 (2.29)	*3.28 (0.51)	*5.14 (1.88)	*3.14 (0.61)	*4.30 (1.76)	*4.02 (1.61)
**^‡^SSIM** Mean (± Std)		0.93 (0.02)	0.87 (0.04)	0.93 (0.02)	0.90 (0.03)	0.91 (0.04)	0.93 (0.02)	0.87 (0.05)	0.93 (0.02)	0.91 (0.03)	0.91 (0.04)
		Original ( x ) – dLVs Reconstructed GCIPL Maps ( $y_{D}$ )					Original ( x ) - dLVs+bLVs Reconstructed GCIPL Maps ( y )				
		Normal	G	ON	NAION	“All”	Normal	G	ON	NAION	“All”
**Annulus** 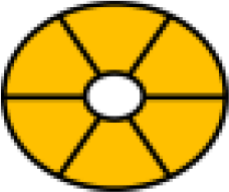		-0.39 (1.07)	0.73 (2.69)	-0.50 (1.24)	0.22 (1.36)	0.04 (1.80)	-0.82 (0.40)	0.17 (1.49)	-0.53 (0.43)	-0.52 (0.58)	-0.40 (0.94)
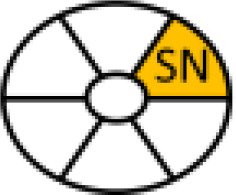	**SN**	0.16 (2.16)	3.21 (6.49)	-0.98 (2.51)	2.09 (3.60)	1.18 (4.45)	-0.07 (0.81)	1.35 (3.27)	0.12 (0.99)	0.04 (1.25)	0.39 (1.98)
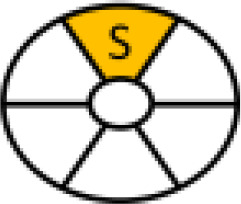	**S**	-0.28 (1.77)	0.18 (3.95)	-0.26 (2.38)	0.28 (2.20)	0.00 (2.74)	-0.76 (1.14)	-0.09 (2.55)	-0.79 (1.26)	-0.76 (1.14)	-0.59 (1.67)
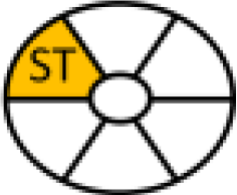	**ST**	-0.29 (2.02)	-1.00 (4.89)	-0.22 (1.90)	-0.11 (2.04)	-0.41 (3.03)	-0.89 (1.19)	-0.38 (2.23)	-0.62 (1.13)	-0.52 (1.26)	-0.58 (1.53)
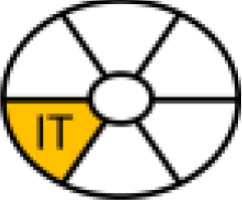	**IT**	-0.51 (1.71)	-0.78 (2.98)	-0.29 (1.72)	-0.94 (2.55)	-0.64 (2.34)	-1.12 (1.09)	-0.51 (2.48)	-1.02 (0.94)	-0.57 (1.34)	-0.78 (1.61)
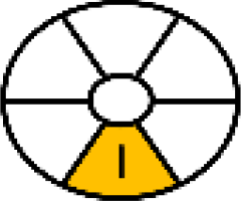	**I**	-0.94 (1.96)	0.44 (4.18)	-0.12 (1.88)	-1.02 (2.51)	-0.37 (2.87)	-1.34 (1.30)	0.10 (2.61)	-0.70 (0.98)	-0.83 (1.39)	-0.64 (1.76)
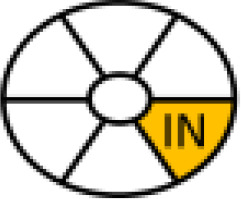	**IN**	-0.49 (2.68)	2.33 (5.78)	-1.12 (2.82)	1.04 (3.89)	0.50 (4.26)	-0.71 (0.99)	0.56 (2.11)	-0.20 (0.95)	-0.49 (1.43)	-0.17 (1.53)

^**†**^RMSE, image-based root-mean-square error (µm); *represents a significant reduction compared to the same disease category (all p -values ≪ 0.001).

^**‡**^SSIM, image-based structural similarity index (33, 34), ranging from 0 (no similarity) to 1 (identical images).

“All”, refers to the data included from all categories (normal, glaucoma, ON, and NAION).

Thickness in Annulus Grid.

Mean Signed Difference (± Std) in Micrometer (µm).

G, glaucoma; ON, optic neuritis; NAION, non-arteritic anterior ischemic optic neuropathy; Thickness in the annulus grid (µm); Annulus, the whole region; SN, superior nasal; S, superior; ST, superior temporal; IT, inferior temporal; I, inferior; IN, inferior nasal.

**Figure 7:**
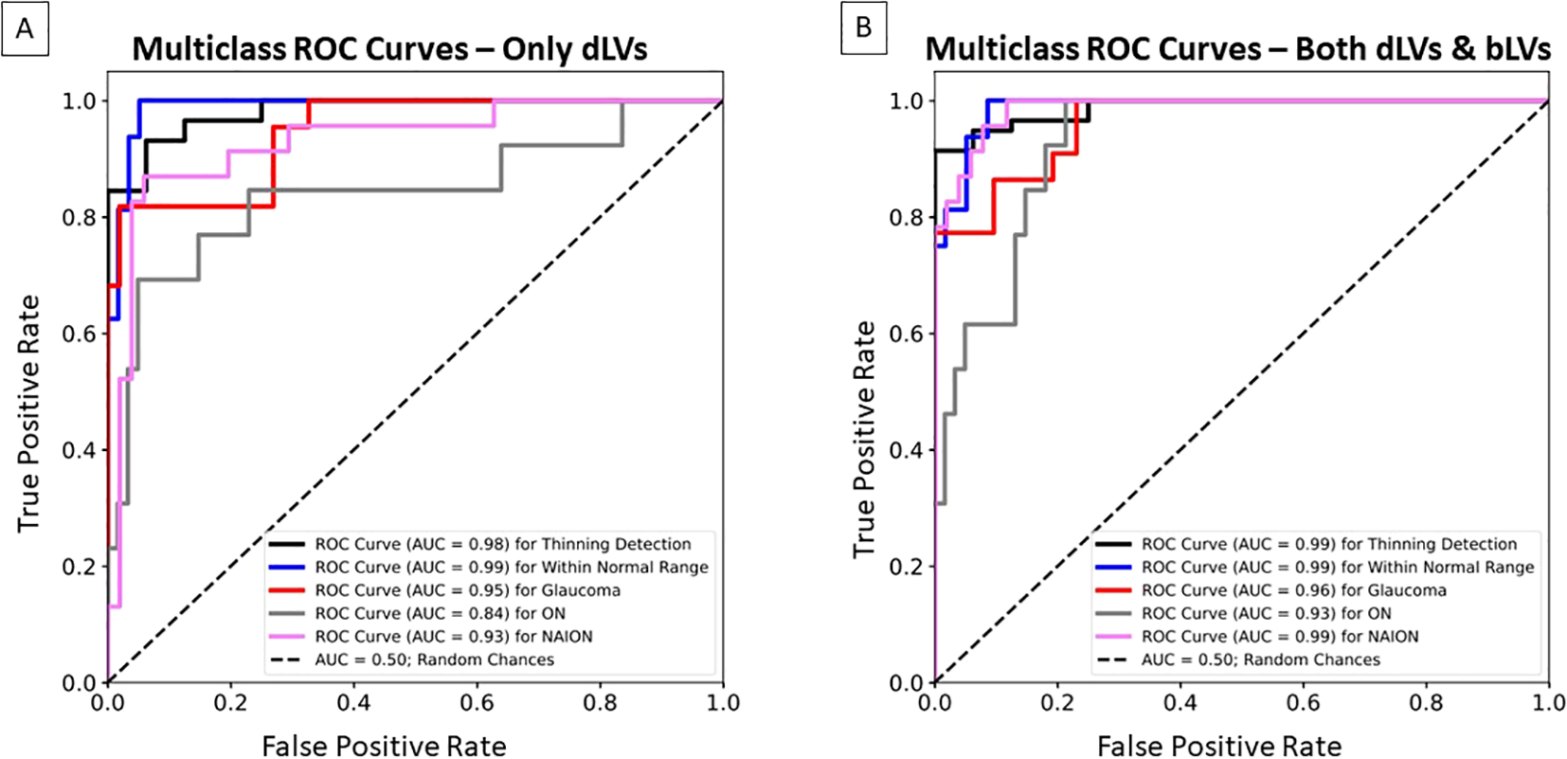
Multiclass Receiver Operating Characteristic (ROC) curves and area under the curve (AUC) for the XGBoost classifier, illustrating the bVAE’s ability to identify GCIPL thinning over time beyond the normal range, within the normal range, glaucoma, ON, and NAION in the independent test dataset. **(A)** The classifier only used the dLVs as input features based on three time points (i.e., the first, middle, and last visits). **(B)** The classifier used both dLVs and bLVs as input features from the same three time points.

**Figures 5**, **6** have shown how dLVs can be used to visualize GCIPL thinning over time through trajectory lines. To further quantify the effectiveness of the latent variables in detecting GCIPL thinning over time, dLVs and bLVs from each eye’s first, second, and third time points were used as features for XGBoost to estimate the thinning probabilities. **Figure 7A** shows the multiclass ROC curves and AUCs based on XGBoost classifiers that used only dLVs from the three time points as features. For the binary classification of thinning beyond the normal range versus within the range, the longitudinal dLVs achieved an AUC of 0.98. However, when distinguishing among specific conditions, the AUCs decreased to 0.95 for glaucoma, 0.84 for ON, and 0.93 for NAION. Incorporating bLVs, which improved the reconstruction errors, as shown in **Table 2**, also considerably enhanced the prediction performance. The AUCs increased to 0.96 for glaucoma, 0.93 for ON, and 0.99 for NAION (**Figure 7B**), highlighting the critical role of bLVs in improving both reconstruction accuracy and predictive ability when incorporating the time course of GCIPL thinning over multiple time points.

## Discussion

This study represents the first application of a VAE approach to display statistically meaningful spatial patterns of RGC loss in glaucoma, ON, and NAION within the GCIPL thickness maps. Unlike conventional global or regional thickness measurements, which use predefined grids for numerical analysis of GCIPL thinning, the proposed bVAE LS montage map introduces a novel, comprehensive perspective. Our previous work (6), limited to 899 OCT macular scans from 25 glaucoma subjects, successfully demonstrated the potential of LS montage mapping to capture glaucomatous RGC loss patterns. In this study, we substantially expanded the dataset to over 10,000 OCT scans from hundreds of patients across the spectrum of optic neuropathies. This larger, more diverse dataset enabled a smoother, more naturally morphing LS montage map (**Figure 3**), providing a refined visualization of RGC loss. Additionally, by integrating residual blocks (19, 29, 35) into the bVAE architecture, the model’s reconstruction capabilities were further enhanced, also contributing to creating more realistic and continuous transitions among patterns in the LS montage map.

Quality control in layer segmentation, particularly regarding verifying fovea location and accurately identifying GCIPL boundary surfaces, is labor-intensive and time-consuming. As current studies increasingly utilize larger datasets, the burden of preprocessing, such as aligning GCIPL thickness maps to the fovea and excluding those apparently off-center images, has become more demanding. Our proposed bVAE model addresses these challenges by bypassing the need for extensive preprocessing. The model is designed to directly reconstruct fovea-centered GCIPL thickness maps while simultaneously providing a probability to identify scans of poor quality, offering a more efficient and convenient way to prepare datasets for use in other large-scale machine learning models. In clinical practice, the bVAE model could also be used to identify poor-quality scans so that they do not significantly impact clinical decisions regarding diagnosis and treatment.

While the bLVs have demonstrated an enhanced encoding of more details from the dLV-reconstructed image ( $y_{D}$ ), **Table 2** shows that the SSIM index for $y_{D}$ is comparable to that of the fully reconstructed image ( y ) in terms of preserving image structure, which is a key feature of tracking RGC loss. This observation is particularly noteworthy, as it raises an important question about the best metrics to evaluate generative models. The primary concern should be capturing meaningful spatial patterns of pathology rather than reconstructing all fine details, such as vessels, minor segmentation inaccuracies, off-centered fovea adjustments, or even epiretinal membrane artifacts (**Figure 6**). Thus, any alternative metrics should better reflect how effectively the model learns relevant features without being distracted by non-essential image characteristics or artifacts. Currently, our bVAE model achieves a promising balance by separating dLVs and bLVs to address both capturing critical spatial patterns and reconstructing finer image details. However, there remains room for exploration to further optimize these components and refine their respective contributions.

The proposed bVAE LS montage map provides advantages over traditional methods like principal component analysis (PCA) by capturing complex, nonlinear spatial patterns of GCIPL thinning within an interpretable latent space. Our previous studies (36, 37) using PCA to analyze Bruch’s membrane shape changes due to increased intracranial pressure indicated the difficulty of displaying all shape combinations in a single 2D morphing map. In contrast, the bVAE’s nonlinear framework organizes statistically meaningful GCIPL spatial patterns using only two display latent variables, enabling smooth transitions across thickening to thinning in various severities. Our bVAE approach supports both intuitive visualization and tracking of disease progression, making the bVAE model a promising alternative for phenotyping optic neuropathies. For instance, the data distributions in the LS montage maps in **Figure 4** visually support the current understanding that glaucoma, ON, and NAION data are more frequently associated with patterns of inferior, diffuse, and superior GCL atrophy, respectively.

Our bVAE model uses the bLVs to capture finer, higher spatial frequency features in the GCIPL thickness maps, allowing the primary dLVs to focus on pathological spatial patterns of optic nerve injury. This design minimizes the influence of mild artifacts (e.g., those from epiretinal membranes [ERMs], major vessels, or minor segmentation errors) on the core disease representations in the LS montage map and dLV reconstructed thickness maps. For instance, **Figure 6** demonstrates how the model used the dLVs to synthesize a reasonable GCIPL thickness map ( $y_{D}$ ) despite the presence of the artifact from ERM. By training with both high-quality and suboptimal GCIPL thickness maps, the bVAE model effectively distinguishes pathological changes from artifacts, enhancing its reliability in tracking optic nerve damage over time across varying image qualities.

When classifying GCIPL thinning over time, the XGBoost classifiers had the lowest AUC for the ON group compared to other groups (**Figure 7**). This can be attributed to the diffuse nature of GCIPL thinning commonly seen in ON, where RGC loss tends to be more widespread. In glaucoma and NAION, damage typically follows the spatial patterns of optic nerve axon bundle loss, making the condition more predictable by our classifiers. This suggests that the bVAE model captures key features relevant to these patterns. In contrast, the progression of GCIPL thinning in ON can vary dramatically due to different factors, such as the severity of demyelinating episodes, the degree of recovery, comorbid conditions like multiple sclerosis, or other causes, such as neuromyeolitic optica. The episodic nature of ON, characterized by such acute vision loss with partial or almost complete recovery, introduces variability in the pattern and timing of RGC layer thinning, making it more challenging for the XGBoost model to classify the ON outcome compared with the thinning due to glaucoma and NAION. In the future, incorporating the actual time between visits (e.g. days) is likely to provide further information that will help differentiate different causes of optic neuropathy that have a time course of thinning that is more specific for that cause. For example, the slower time course of progression in glaucoma *vs*. NAION or ON or compressive optic neuropathy can be used to better classify disease in addition to the spatial pattern of nerve loss. In addition, incorporation of bVAE models using spatial patterns of retinal nerve fiber layer (RNFL) thickness, optic disc morphology and visual field loss provide a multimodal approach to classification of disease and monitoring of treatment.

There are a few limitations to this study. The age distributions of subjects differed among the three diseases due to their distinct pathophysiological profiles; ON patients were generally younger, NAION patients were older, and glaucoma patients represented the oldest cohort in this study. A notable strength of the NAION dataset is that it originated from the Quark NAION study (31), which included participants within 14 days of symptom onset, allowing for the capture of the acute phase of the disease. However, the timing of visits in the Quark study OCT imaging protocol could not be matched to the glaucoma and ON datasets due to differences in data collection protocols and timelines. In the future, we intend to incorporate the actual time between visits into the prediction model and include progression rates across diseases. This approach will enable us to further assess how latent variables’ movement within the latent space reflects disease progression, enhancing bVAE model’s clinical relevance.

Another limitation of this study is the absence of additional clinical parameters such as mean deviation (MD) from visual fields, intraocular pressure (IOP), visual acuity, and mean peripapillary RNFL (pRNFL) thickness, which can impact staging and tracking the clinical course of optic neuropathies. However, this study focuses on developing a novel approach to visualize and quantify spatial patterns in data using bVAE latent variables and latent space. Clinical data will eventually be added as labels, but its absence here does not compromise the primary significance of this work, which lies in the visualization and quantification of spatial patterns of nerve loss, providing additional insights into traditional OCT global metrics, like the average pRNFL thickness. We are currently applying the bVAE to quantify spatial patterns of visual field test results in latent space so that both structure (OCT) and function (visual field) can be visualized and monitored over time in a similar way.

Additionally, the determination of whether GCIPL thinning progressed beyond or remained within the normal range over time was based on visual inspection by an OCT expert (J-KW). While the primary aim was to demonstrate that the bVAE’s latent variables could effectively capture key spatial patterns of GCL thinning, future work could further refine these subjective labels by using linear regression to determine GCIPL thinning status and incorporating clinical diagnoses supported by additional parameters or evidence (e.g., MD, IOP, visual acuity, etc.)

A further limitation of this study is the need for external dataset validation to confirm the generalizability of the proposed bVAE model. Although the mixed dataset in this study comprises 10,710 OCT scans from 822 subjects across multiple sources (including the Quark clinical trial, New York Mt. Sinai Hospital, and the University of Iowa) further testing on new, independent datasets would provide additional support for the model’s robustness across diverse populations. Validating the bVAE model with external data would also enhance its utility in varied clinical environments and ensure that the latent space organization remains consistent for broader applications.

In summary, this study introduces a novel approach for visualizing the pattern and severity of RGC loss by utilizing latent variables to capture key spatial patterns associated with different causes of optic nerve disease and its progression. More future work by our group will involve incorporating additional data formats, such as OCT RNFL thickness maps, optic disc morphology, visual field measurements, and OCT angiography, further enhancing the model’s ability to visualize, analyze, and demonstrate disease diagnosis and progression as well as treatment effects. The unsupervised nature of the bVAE model allows for flexibility and adaptability to different clinically relevant parameters, making it a highly versatile tool for future applications.

## Data Availability

The raw data supporting the conclusions of this article will be made available by the authors, without undue reservation.
