# Supplementary material for: Quantifying the spatial patterns of retinal ganglion cell loss and progression in optic neuropathy by applying a deep learning variational autoencoder approach to optical coherence tomography
Source: Front Ophthalmol (Lausanne). 2025 Feb 3;4:1497848. doi: 10.3389/fopht.2024.1497848 (PMC11830743; doi:10.3389/fopht.2024.1497848)
Supplement: Supplementary file 1 [file DataSheet1.pdf]

Supplemental Document

Methods – Booster Variational Autoencoders (bVAEs)

We developed a booster variational autoencoder (bVAE) as an extension of our previous bi-channel VAE used for analyzing optic nerve "C-shape" swelling in papilledema[1]. To capture the more complex patterns of retinal ganglion cell (RGC) loss in ganglion cell inner plexiform layer (GCIPL) thickness maps, we introduced booster latent variables (bLVs) to supplement the information provided by the display latent variables (dLVs, i.e., d1 and d2). The bVAE architecture comprises an encoder (E), a display decoder (D_D), and booster decoder (D_B); all constructed using convolutional residual blocks to enhance gradient flow during backpropagation, as demonstrated in prior studies[2, 3]. The encoder E processes the input GCIPL thickness map (x) to compute latent variables (d′1, d′2, LogVar1, LogVar2, b1, b2, ⋯, b8), estimate the fovea location (f_x, f_y) and flag images with poor quality (f_{EXC}), shown in Fig. 2.

To increase variability in the dLVs, bVAE includes normalized Gaussian sampling units and utilizes the reparameterization trick[4] for enabling gradient-based optimization between E and D_D. We applied Kullback-Leibler (KL) divergence to constrain dLVs and normalized Gaussian distributions to enforce smoothness in the display latent space. The display decoder D_D uses the dLVs to reconstruct a fovea-centered version of x, denoted as x_C, resulting in an output y_D that captures the primary GCIPL spatial patterns. The bLVs (i.e., b1, b2, ⋯, b8) are directly output from E and concatenated with the dLVs as input to the booster decoder D_B, which estimates the deviation between y_D and x_C, producing a deviation map y_B. The final reconstructed GCIPL thickness map (y) is obtained by adding the deviation map (y_B) to the dLV-reconstructed image (y_D).

The overall loss function ($\mathcal{L}$) consisted of four main components: the image reconstruction errors ($\mathcal{L}_{Rec}$), reconstruction quality assessment ($\mathcal{L}_{QA}$), KL divergence ($\mathcal{L}_{KL}$), and display constraint ($\mathcal{L}_D$). $\mathcal{L}_{Rec}$ penalizes differences between the input and reconstructed images using mean square error (MSE) measurements. First, we calculate $MSE(x_C, y_D)$ and $MSE(x_C, y)$ as the basic reconstruction errors. To enhance regional information in areas sensitive to early optic nerve axon bundle defects, we define a rectangular region of interest (ROI) on the temporal side of the fovea, covering 0.9 mm in width and 1.8 mm in height. Then, we calculate $MSE(x_{C,ROI}, y_{D,ROI})$ as the regional reconstruction penalty. The final $\mathcal{L}_{Rec}$ is formatted as

$$\mathcal{L}_{Rec} = \beta_{Rec,D} \times MSE(x_C, y_D) + \beta_{Rec} \times MSE(x_C, y) + \beta_{Rec,ROI} \times MSE(x_{C,ROI}, y_{D,ROI}), \quad (1)$$

Where $\beta_{Rec,D}$, β_{Rec}, and $\beta_{Rec,ROI}$ are the weighting coefficients for each term.

For the reconstruction quality assessment, we use a wavelet-based haar perceptual similarity index[5] (HarrPSI) and gradient magnitude similarity deviation[6] (GMSD) for quantification. Both implementations can be found in the PyTorch Image Quality Assessment package[7]. Since y_D often requires more regional attention, we focus on $Haar(x_{C,ROI}, y_{D,ROI})$ and $GMSD(x_{C,ROI}, y_{D,ROI})$. The final $\mathcal{L}_{QA}$ is defined as

$$\mathcal{L}_{QA} = \beta_{Haar} \times Haar(x_{C,ROI}, y_{D,ROI}) + \beta_{Haar} \times Haar(x_C, y) + \beta_{GMSD} \times GMSD(x_{C,ROI}, y_{D,ROI}) + \beta_{GMSD} \times GMSD(x_C, y), \quad (2)$$

where β_{Haar} and β_{GMSD} are the weighting coefficients for each term.

Next, the KL divergence penalizes the dissimilarity between the encoder latent variable distribution $E(\boldsymbol{d}|x)$ and standard Gaussian distribution ($N(0,1)$), where $E(\cdot)$ represents the function of the encoder, and $\boldsymbol{d}$ represents the distribution of the dLVs. The KL-divergence in our design is defined as

$$\mathcal{L}_{KL} = \beta_{KL} \times KL(E(\boldsymbol{d}|\boldsymbol{x}), N(0,1)), \quad (3)$$

Where β_{KL} is the weighting coefficient for KL divergence. We assume that $E(\boldsymbol{d}|\boldsymbol{x})$ follows a normal distribution, so $E(\boldsymbol{d}|\boldsymbol{x})$ can also be represented as $N(\boldsymbol{\mu}, \boldsymbol{\sigma})$, where $\boldsymbol{\mu}$ = (d′1, d′2) and $\boldsymbol{\sigma}$ = (LogVar1, LogVar2).

In order to encourage the dLVs to be associated with the morphing patterns of the RGC axon bundle defects, we impose constraints based on ratios of regional GCIPL thickness according to the Zeiss Cirrus annulus grid sectors. Specifically, the loss term $\mathcal{L}_D$ is defined as

$$\mathcal{L}_D = \beta_D \times \left(d1 - c \times (T_{IN} + T_I + T_{IT})\right) + \beta_D \times \left(d2 - c \times (T_{SN} + T_S + T_{ST})\right), \quad (4)$$

Where β_D is the weighting coefficient for the display constraint, c is a scaling constant, and T_{IN}, T_I, T_{IT}, T_{SN}, T_S, and T_{ST} represent the sector thicknesses in x at the inferior-nasal, inferior, inferior-temporal, superior-nasal, superior, and superior-temporal regions, respectively.

Overfitting

To reduce the risk of model overfitting, we leveraged the VAE's inherent design, allowing both visual and quantitative monitoring. The VAE latent space montage map serves as a built-in visual inspection tool, as overfitting would appear as non-anatomical or irregular GCIPL defect patterns. Additionally, we tracked reconstruction errors as an objective metric of model performance, observing minimal differences in the clean test dataset (see Table 2), which supports the model's generalizability. The use of a large training set (8,931 GCIPL thickness maps) and convolutional residual blocks[2, 3] further reduced the overfitting risk. Although these measures have proven effective, future validation on larger, external datasets will further enhance the model's robustness.

Methods – Data Quality Control & Correction of Fovea Location

For each OCT macular scan, a graphical report (Sup. Fig. 1) was generated post-segmentation, containing multiple sections to display: (1) horizontal and vertical B-scans with layer segmentation, (2) *en-face* images of the inner limiting membrane (ILM), the junction surface between the retinal nerve fiber layer (RNFL) and the retinal pigment epithelium (RPE) complex, (3) thickness maps of the RNFL, GCIPL, inner nuclear + outer plexiform layer (INOPL), outer nuclear layer plus photoreceptors (ONL and below), and the total retina, and (4) corresponding deviation maps highlighting areas of thinning (yellow/red superpixels below the 5th percentile) and thickening (purple superpixels above the 95th percentile) compared to our Iowa normative dataset. We also developed a review system to efficiently support quality control, including adding notes and correcting the automatically identified fovea location. The figures below are a few examples.

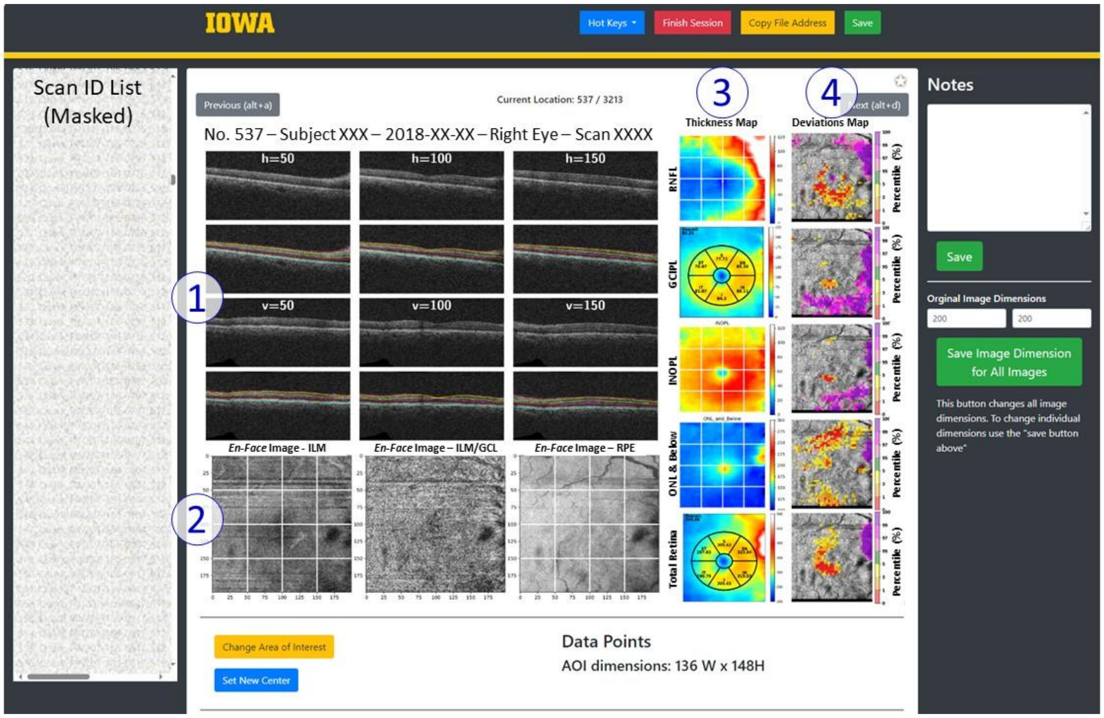

Sup. Fig. 1 Representative OCT scan that passed quality control. This report demonstrates correct layer segmentation and accurate fovea localization, meeting the criteria for inclusion in the analysis.

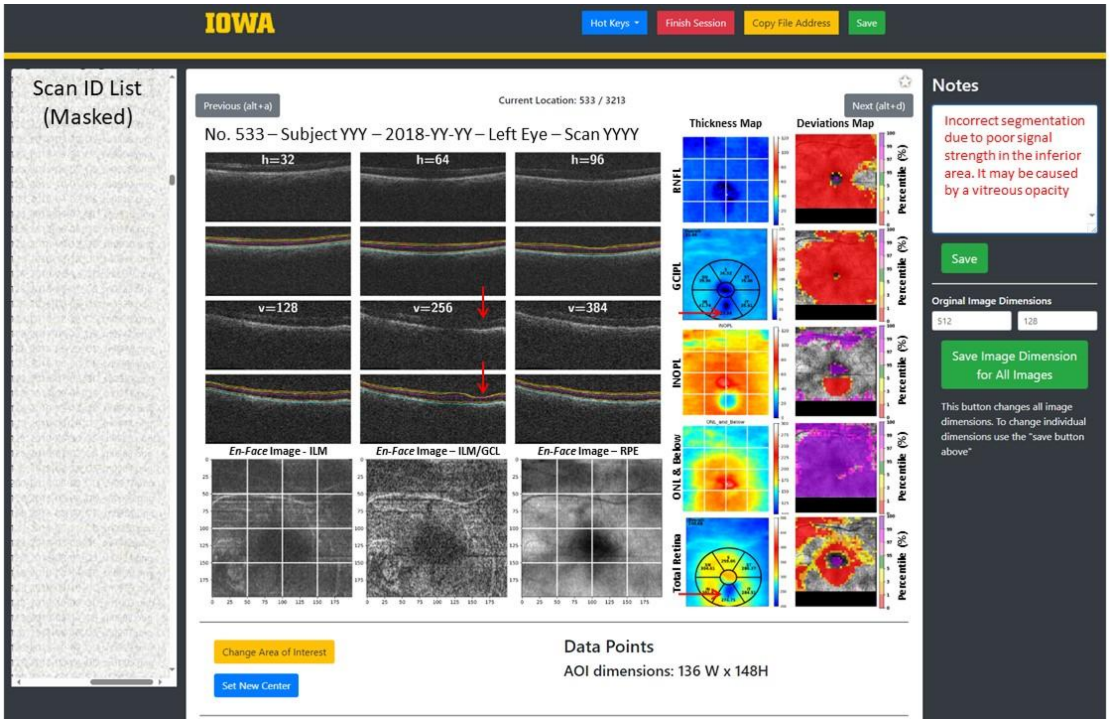

Sup. Fig. 2 Example of an OCT scan flagged for incorrect layer segmentation in the inferior sector. Errors are observed in the GCIPL and total retina thickness maps, as indicated (the red arrows).

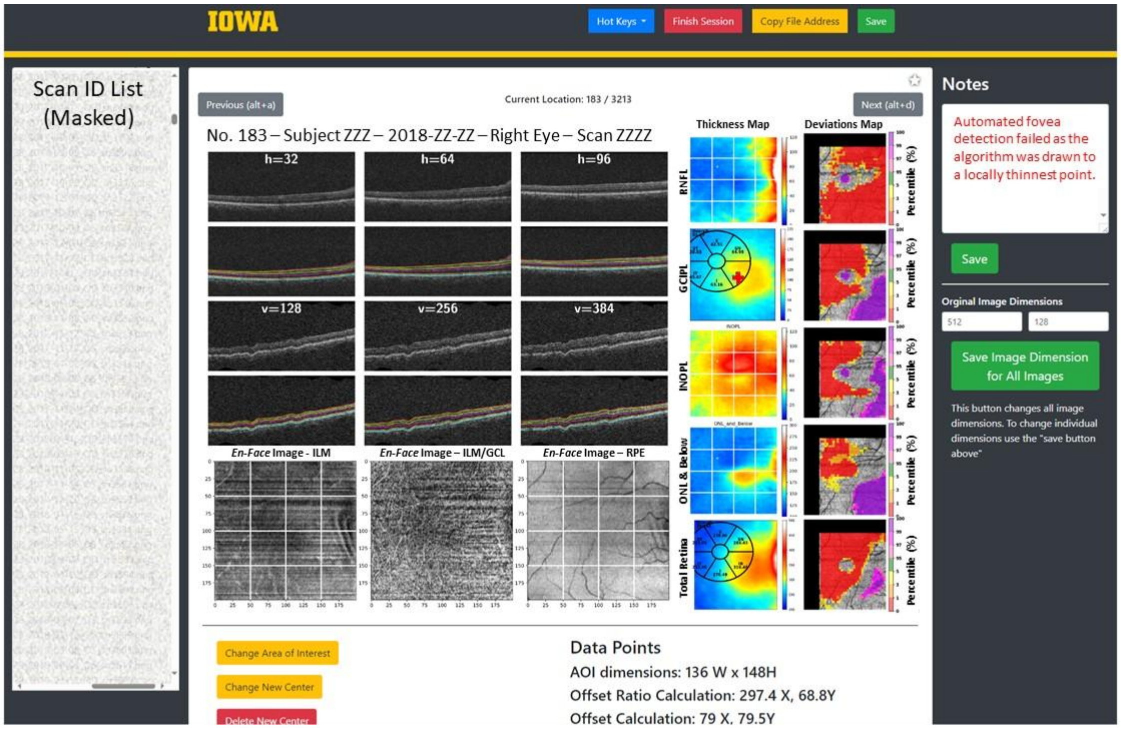

Sup. Fig. 3 Example of an OCT scan flagged for incorrect fovea location. A manually corrected fovea location is indicated by the red cross.

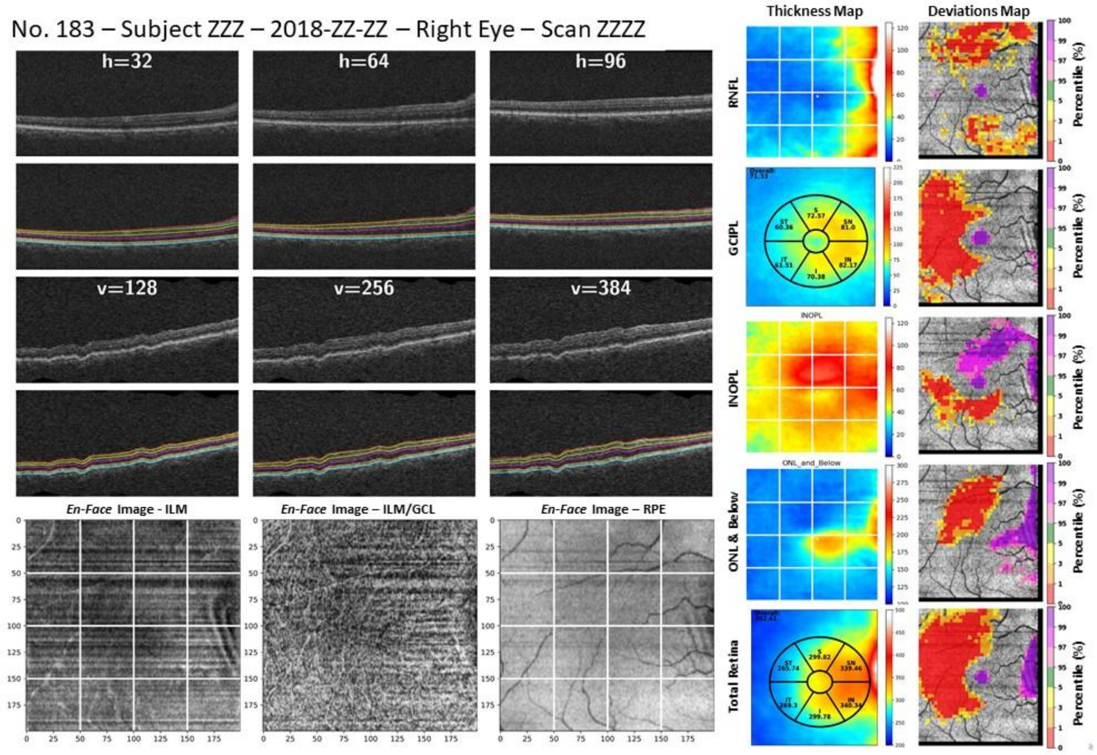

Sup. Fig. 4 Quality control report showing the corrected fovea location for the same case presented in Fig. 3.

Manual Assessment of Longitudinal Change

For each subject, GCIPL thickness maps were organized by visit and included both eyes together. By qualitatively assessing changes in GCIPL spatial patterns to a normal range between both eyes and over time, each eye was then assigned one of two labels: Thinning or Not-Thinning.

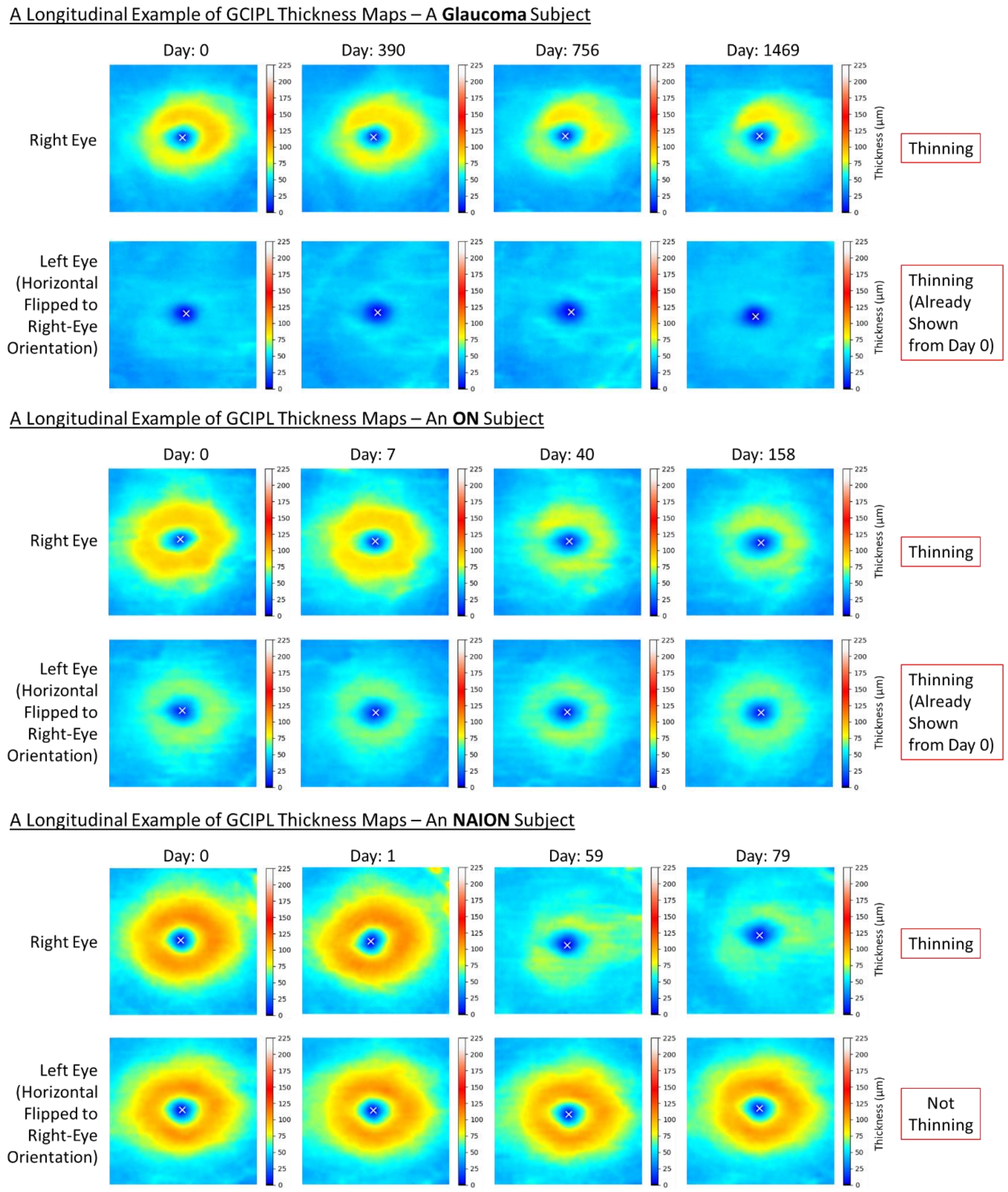

Sup. Doc. Fig. 5 Longitudinal GCIPL changes in representative cases of glaucoma, ON, and NAION. Day 0 represents the day the patient first underwent OCT imaging.

References

1. Wang J-K, Linton EF, Johnson BA, et al. Visualization of optic nerve structural patterns in papilledema using deep learning variational autoencoders. *Translational Vision Science & Technology* 2024;13:1-13.
2. Daniel T, Tamar A. Soft-introvae: Analyzing and improving the introspective variational autoencoder. *2021 IEEE/CVF Conference on Computer Vision and Pattern Recognition (CVPR)*. Virtual; 2021:4391-4400.
3. Huang H, Li Z, He R, Sun Z, Tan T. Introvae: Introspective variational autoencoders for photographic image synthesis. *Neural Information Processing Systems (NeurIPS)*; 2018:1-12.
4. Kingma DP, Welling M. An introduction to variational autoencoders. *Foundations and Trends in Machine Learning* 2019;12:307-392.
5. Reisenhofer R, Bosse S, Kutyniok G, Wiegand T. A haar wavelet-based perceptual similarity index for image quality assessment. *Signal Processing: Image Communication* 2018;61:33-43.
6. Xue W, Zhang L, Mou X, Bovik AC. Gradient magnitude similarity deviation: A highly efficient perceptual image quality index. *IEEE Transactions on Image Processing* 2014;23:684-695.
7. Rozet F. Piqa: Pytorch image quality assessement. Version: 1.2.0; 2020.
